# Development of Biocompatible Ciprofloxacin–Gold Nanoparticle Coated Sutures for Surgical Site Infections

**DOI:** 10.3390/pharmaceutics14102130

**Published:** 2022-10-07

**Authors:** Sunitha Sampathi, Pankaj Kumar Tiriya, Sujatha Dodoala, Vijayabhaskarreddy Junnuthula, Sathish Dyawanapelly

**Affiliations:** 1 GITAM School of Pharmacy, GITAM (Deemed to be University), Hyderabad 502329, India; 2 Department of Pharmaceutics, National Institute of Pharmaceutical Education and Research (NIPER), Balanagar, Hyderabad 500037, India; 3 Institute of Pharmaceutical Technology, Sri Padmavati Mahila Viswavidyalayam, Tirupati 517502, India; 4 Drug Research Program, Faculty of Pharmacy, University of Helsinki, Viikinkaari 5 E, 00790 Helsinki, Finland; 5 Department of Pharmaceutical Science and Technology, Institute of Chemical Technology, Mumbai 400019, India

**Keywords:** gold nanoparticles, sutures, surgical site infections, ciprofloxacin, biocompatibility

## Abstract

Surgical site infections (SSIs) are mainly observed after surgeries that use biomaterials. The aim of this present work was to develop ciprofloxacin hydrochloride (CPH)-loaded gold nanoparticles. These ciprofloxacin–gold nanoparticles were coated onto a sterile surgical suture using an adsorption technique, followed by rigidization via ionotropic crosslinking using sodium alginate. Furthermore, UV-visible spectroscopy, infrared spectroscopy, and scanning electron microscopy were used to characterize the samples. The particle size of the nanoparticles was 126.2 ± 13.35 nm with a polydispersity index of 0.134 ± 0.03, indicating nanosize formation with a monodispersed system. As per the International Council for Harmonization of Technical Requirements for Pharmaceuticals for Human Use (ICH) guidelines, stability studies were performed for 30 days under the following conditions: 2–8 °C, 25 ± 2 °C/60 ± 5% RH, and 40 ± 2 °C/75 ± 5% RH. For both Gram-negative and Gram-positive bacteria, the drug-coupled nanoparticle-laden sutures showed a twofold higher zone of inhibition compared with plain drug-coated sutures. In vitro drug release studies showed a prolonged release of up to 180 h. Hemolysis and histopathology studies displayed these sutures’ acceptable biocompatibility with the healing of tissue in Albino Swiss mice. The results depict that the use of antibiotic-coated sutures for preventing surgical site infection for a long duration could be a viable clinical option.

## 1. Introduction

Polymeric biomaterials have attracted the interest of medical researchers in recent years. One of the important applications of these biomaterials is in the field of medical devices, such as implants [1,2], sutures, catguts, ligatures, gauzes [3,4], antimicrobial polymers [5], hydrogels [6], and nanofibers [7]. Surgical sutures are used to sew an incision and close a wound to prevent pathogens from infecting the wound and enhance its healing [8]. Surgical sites are highly prone to nosocomial infections [9]. Surgical site infections (SSIs) result in high mortality rates, and the treatment is expensive [10,11,12]. Generally, after surgery, an SSI prolongs illness, leading to death in patients. Out of 27 million operations in the United States, approximately 2–5% led to SSIs, culminating in approximately 300,000–500,000 cases annually [13,14]. The Vicryl Plus Antimicrobial Suture (VPAS) is currently marketed as having triclosan as its principal component. However, it was reported that triclosan is deposited in breast milk, umbilical cord serum, fatty tissue, and urine. It may interfere with the immunogenic, hormonal, and copulatory functions of the body, causing serious adverse effects [15,16]. Therefore, appropriate substitutes are urgently needed. Recently, the application of drug-conjugated sutures with several antimicrobial agents was reported to reduce SSIs [17,18,19]. The surface coating of sutures and catguts with broad-spectrum antibiotics may prohibit the colonization of pathogens without altering the physical characteristics of the suture and catgut [20,21].

The evolution of nanotechnology was shown to be a promising approach to formulating some conventional systems to nanoparticulate systems that exhibit new physiological and biomedical properties [22,23,24,25,26,27]. Among the metallic nanoparticles [28], gold nanoparticles (G-NPs) have been studied intensively by researchers in recent years. G-NPs combined with antimicrobials were found to produce an enhancement in antibacterial activity [29,30]. G-NPs are biologically inert [31]; can be synthesized in various sizes and shapes [32,33]; have the ability to effectively bind to drugs via electrostatic, covalent, or noncovalent interactions [34]; and are capable of site targeting [25,26,35,36]. The other advantages are the possibility of scale-up [37,38]; the ability to control the bioavailability, biodistribution, and biopharmacokinetics of entrapped molecules [39]; overcoming multidrug resistance (MDR) against various bacteria [40]; and so on. Metallic nanoparticles have a large surface area because of their small size, enhancing their biological and chemical activity [28,41]. Antibiotics in the form of nanoparticles decrease unfavorable drug effects on cells by reducing the drug dose but also improve their antibacterial properties at the bioactive site [42,43]. Here, we developed surgical sutures with ciprofloxacin (broad-spectrum antibiotic)-loaded gold nanoparticles to prevent SSIs. In the present study, we employed polyvinylpyrrolidone (PVP) to stabilize the gold NPs. PVP addition is known to increase the stability of gold NPs because of its high surface-active properties and solubility in aqueous media [44]. We evaluated the antibacterial activity against *E. coli* (a Gram-negative bacteria) and *B. subtilis* (a Gram-positive bacteria) and further demonstrated the biocompatibility of ciprofloxacin–gold nanoparticle-fabricated surgical sutures.

## 2. Materials

Ciprofloxacin hydrochloride (CPH) was a generous gift from Nitin Life Sciences, Himachal Pradesh, India. Aurochloric acid, polyvinyl pyrrolidone (mol. wt. 40,000 Da), sodium tetra borohydride, sodium alginate, and trisodium citrate were purchased from Sigma Aldrich, Karnataka, India. TruglyudeTM Fast was obtained from Sutures India (SPIL, Bengaluru, Karnataka, India). Potassium dihydrogen orthophosphate and calcium chloride were purchased from Loba Chemie, Mumbai, India. Nutrient agar, trypticase soya broth, and the bacterial strains *Escherichia coli* (ATC 25922) and *Bacillus subtilis* (ATCC 6633) were purchased from Himedia, Mumbai, India. All the other reagents utilized were of analytical grade.

## 3. Methods

### 3.1. Preparation and Evaluation of Ciprofloxacin–Gold Nanoparticles

Ciprofloxacin-loaded gold nanoparticles were prepared by using the method previously reported by Gangwar et al. with slight modifications [45].

#### 3.1.1. Formulation of Gold Nanoparticles (G-NPs)

In summary, a 5 mM solution of aurochloric acid was made, and 1 mL was added to 10 mL of double distilled water while stirring continuously at 300 rpm for 1 h (80 °C). Later, 2 mM sodium tetra borohydride solution (2 mL) was added and agitated further for the formation of gold nanoparticles (G-NPs), as indicated by a color change from colorless to wine red [45].

#### 3.1.2. Polyvinyl Pyrrolidone (PVP)-Capped G-NPs (PG-NPs)

In brief, polyvinyl pyrrolidone (PVP; 100 mg) was added to 10 mL of gold nanoparticle solution (1% *w*/*v*) and stirred for 12 h. The unconjugated PVP was separated using the dialysis bag method (molecular weight 12,000 Da) by suspending it in 40 mL of distilled water for 4 h with continuous stirring.

#### 3.1.3. Ciprofloxacin-Loaded PVP Capped Gold Nanoparticles (CPG-NPs)

For the formation of the ciprofloxacin-loaded polyvinyl pyrrolidone-capped gold nanoparticles, 30 mg of drug dissolved in double-distilled water (1 mL) was added to the above solution and stirred on a magnetic stirrer (300 rpm) at room temperature for 3 h. The nanoparticles were collected via centrifugation (5000 rpm for 30 min).

#### 3.1.4. UV-Visible and FT-IR Analysis

By examining the plasmon resonance of metallic nanoparticles in the 200–800 nm region, the absorbance of G-NPs, PG-NPs, and CPG-NPs was studied using a UV spectrophotometer (Jasco; Model: V-650). Additionally, the spectra in the region of 400–4000 cm^−1^ were obtained using an FT-IR spectrometer (Perkin Elmer, Waltham, MA, USA) [46,47].

#### 3.1.5. Particle Size, Polydispersity Index (PDI), and Zeta Potential

The particle size and polydispersity index (PDI) of the synthesized G-NP, PG-NP, and CPG-NP formulations were measured. The samples were diluted ten-fold with distilled water and analyzed using a Malvern Zetasizer, Malvern, UK (ZS90, UK). The zeta potential was measured using laser Doppler velocimetry as an average of 10 measurements [48,49,50].

### 3.2. Preparation and Evaluation of CPG-NP-Coated Sutures

The sodium alginate ciprofloxacin colloidal gold nanoparticles were then coated onto surgical sutures (TRUGLYDE Fast) using the slurry dipping technique in an aseptic chamber. Briefly, ciprofloxacin–gold nanoparticles were added to a sodium alginate solution (2% *w*/*v*) under constant mixing on a magnetic stirrer at 150 rpm; later, they were stored at 25 °C for 8 h until further use. The surgical suture (3 cm length) was immersed in the above coating solution for different time intervals: 30, 60, 90, and 120 min. Finally, the sutures were crosslinked with 4% *w*/*v* calcium chloride solution for 15 min [51].

#### 3.2.1. Surface Morphology

The surface topology of gold nanoparticles and coated and plain sutures (PSs) was determined using SEM (Hitachi High-Tech Corporation, Tokyo, Japan S-3700N) at a voltage of 30 kV with 500–10,000× magnification [52,53].

#### 3.2.2. In Vitro Release Studies

In vitro drug release from the coated sutures was performed by immersing the sutures in a flask containing simulated body fluid (SBF; 50 mL) whose pH was 5 (to mimic the acidic conditions of an inflamed site), as reported by Marques et al. [54]. The flask was tightly sealed via a sterile cotton plug to prohibit any environmental contamination. The flask was stirred at 150 rpm at 37 ± 0.5 °C on a shaker. At prefixed periods (0.5–184 h), 5 mL of the sample was withdrawn, replaced with fresh medium, and analyzed for drug content using UV-vis spectroscopy (Supplementary Materials Method S1 and Figure S5) at 275 nm.

#### 3.2.3. Determination of Tensile Strength (TS) and Elongation at Break (E/B)

The diameters of the plain and coated sutures were assessed using a micrometer at various points along the sutures; the average diameter was determined and represented in millimeters. The Ultratest (Mascesin, UK) set with a 25 kg load cell was used to test two important mechanical characteristics, namely, TS and E/B, with minor modifications from prior publications [55]. In brief, an 8 cm length of coated suture was placed between two clamps spaced 3 cm apart, and the strips were tugged at a rate of approximately 1 mm/s with the top clamp until the film broke. TS and E/B were then calculated.

#### 3.2.4. Stability Studies

To check the integrity and stability of drug-loaded gold-nanoparticle-coated sutures, studies were conducted for 30 days under different conditions (2–8 °C, 25 ± 2 °C/60 ± 5% RH, and 40 ± 2 °C/75 ± 5% RH) as per ICH guidelines [56,57]. The sutures were evaluated for their drug content, cumulative drug release, and similarity factor for the drug release values. The similarity factor was calculated to determine the change in the release pattern, which may be an indication of the instability of the conjugation during storage in adverse conditions and is calculated using the equation below:

(1) $$\mathrm{Similarity}\;\mathrm{factor}\;(f2)=50\log\;[\surd \{1+1/n\sum_{r=1}^{n}\left(Rt-Tt\right)\}]\times 100\;$$

where *n* is the number of time points where samples were withdrawn, *R_t_* is the reference sample or sample at 0 days, and *T_t_* is the sample at 30 days at each time point.

#### 3.2.5. Antibacterial Activity

The antibacterial activity of the various sutures (coated and uncoated) was determined in *Escherichia coli* (a Gram-negative bacteria) and *Bacillus subtilis* (a Gram-positive bacteria) using the disc diffusion technique [58]. Bacterial strains were swabbed on nutrient agar plates using sterile swabs. The test organisms were grown in nutrient broth overnight at 37 °C for 24 h. Ciprofloxacin-coated sutures, CPG-NP-coated sutures (2 cm in length), and normal saline solution (as a negative control) were placed on nutrient agar medium plates. The Petri dishes were incubated for 72 h at 37 °C, and the zone of inhibition was determined. Studies were performed in triplicate.

#### 3.2.6. Hemolysis

The study was performed as per an earlier report [51]. Plain sutures, ciprofloxacin-coated sutures, and CPG-NP-coated sutures of 2 cm dimensions were kept in 0.9% *w*/*v* sodium chloride solution for 24 h at 37 ± 0.5 °C in biological oxygen demand (BOD). The blood sample (250 µL) was homogeneously admixed with the respective test samples in a sterile vial and incubated for 3 h at room temperature, and the absorbance was checked at 540 nm via a microplate ELISA reader (Benesphera E21). Sodium carbonate (0.1% *w*/*v*) and normal saline of pH 7.2 were used as positive and negative controls, respectively. By using the equation below, the percentage of hemolysis was calculated:

(2) $$\mathrm{HP}\;(\%)=\frac{\left(d-b\right)}{\left(\mathrm{Dp}-\mathrm{Dn}\right)}\times 100$$

where d is the absorbance of the test, Dp is the positive control absorbance output, b is the blank, and Dn is the negative control absorbance output.

#### 3.2.7. Histopathology

The Institutional Animal Ethics Committee (IAEC) approved the animal study protocol (NIP/2/2015/PE/132). Prior to the investigation, albino mice (20–30 g) aged 4–5 weeks were incubated in light/dark settings for a week. The animals were divided into four groups (*n* = 8): Group I acted as a negative control group, and intestinal anastomosis was not performed in this group. In groups II, III, and IV, the mice were anesthetized with ketamine (50 mg/kg, intramuscular) and xylazine (5 mg/kg, intramuscular) before cutting (2 cm) the ileum near the cecum with sterile scissors. Then, PS, CPH-coated sutures, and CPG-NP-coated sutures were used to close the wound, resulting in interrupted anastomosis in groups II, III, and IV, respectively. This was followed by the closure of the peritoneal and skin layers of the abdominal wall with a normal suture. Following surgery, the mice were given free access to water and liquid food. After one week, the animals were euthanized, the ileum of the animals was excised, and adhesion development scores were measured. The adhesion values were calculated using the Vander Hamm et al. scale [59]. Isolated samples (ileum) were preserved in formalin until further analysis. Under a light microscope, histological sections of the ileum were evaluated in a blinded fashion for criteria such as inflammatory cell interruption and any fibroblast proliferation using the revised Ehrlich and Hunt numerical scale (0–4) [60]. The entire procedure was performed in an aseptic sterile chamber.

#### 3.2.8. Statistical Data Analysis

The mean ± standard deviation of the results was calculated, with ‘n’ being the number of samples studied. Using Prism software, Student’s *t*-test was used to calculate the differences between the means (version 6.01; Graph Pad, San Diego, CA, USA), and *p* ≤ 0.05 was considered statistically significant.

## 4. Results and Discussion

### 4.1. Characterization of CPG-NPs

**Figure 1:**
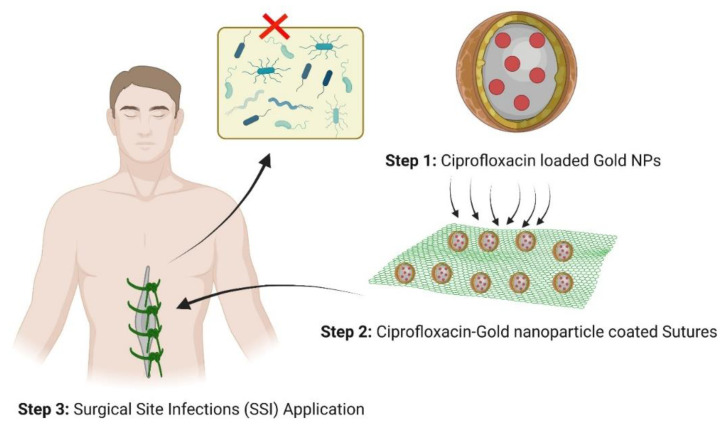
Schematic representation of biocompatible ciprofloxacin–gold-nanoparticle-coated sutures for surgical site infections.

In the present study, gold nanoparticles were synthesized by adding 2 mM sodium tetra borohydride to 5 mM aurochloric acid at a ratio of 1:1 as a reducing agent and as a metal precursor. The development of CPG-NPs was accomplished in two steps. PVP (1% *w*/*v*) was coated onto the optimized gold nanoparticles on a magnetic stirrer in the first stage to make PG-NPs, which was confirmed by analyzing UV spectra. UV-visible spectra of G-NPs, PVP-conjugated G-NPs, ciprofloxacin, and CPG-NPs in aqueous media are shown in Supplementary Materials Figure S1. PVP conjugation on gold nanoparticles led to a characteristic absorption plasmon resonance band observed at 548.63 nm (yellow band). The absorption in the 200 to 400 nm region showed distinct bands at 273.2 and 322.2 nm due to the absorption of the ciprofloxacin molecule, as represented by the dark blue band. The λ_max_ at 273.8 nm relates to the fluorobenzene moiety’s π-π transition, while the other corresponds to the quinolone ring’s n-π transitions. The solution consisting of PVP-capped drug-loaded gold nanoparticles had a deep and characteristic dark green color that occurred due to plasmon absorption at 543.41 nm. This indicated a bathochromic shift of ciprofloxacin’s plasmon resonance peak from 273.2 to 307.2 nm. Thus, the UV-visible data supported claims of IR spectra results showing that ciprofloxacin was attached via hydrogen bonding to the gold nanoparticle surface with the help of PVP [45]. The representative scheme is represented in Figure 1.

FT-IR spectroscopy was performed to verify the occurrence of interactions between the drugs and polymers. The infrared spectra of the G-NPs, PG-NPs, CPH, CPG-NPs, and the physical mixture are shown in Supplementary Materials Figure S2. The signatures of CPH were free OH groups (3447 cm^−1^), as well as alkenes and aromatic C-H stretching (2711 cm^−1^). The double-bonded functional group at 2081 cm^−1^ and a bond at 1636 cm^−1^ were due to pyridine moieties, and the absorption band at 1272 cm^−1^ was attributed to C–N stretching. The spectrum verified the conjugation of NaBH_4_-reduced gold nanoparticles with PVP via a blueshift from 1642 cm^−1^ to 1637 cm^−1,^ which may have been due to intermolecular hydrogen bonding. The distinctive stretching vibration of the C–H bond was responsible for a bond at 2925 cm^−1^. At 1637 cm^−1^, a vibration bond of the C=O group occurred, indicating that PVP contained bonding carbonyl groups. The spectra showed the linkage of CPH with PG-NPs via intermolecular hydrogen bonding to the enolic hydrogen group, which may have led to –O–H broadening at 3446 cm^−1^ compared with G-NPs. The peaks produced by the optimized formulations correlated with the drug spectra peaks. This indicated that the drug retained its identity after processing with the formulation components. Hence, it can be concluded that molecular interactions did not occur that could have altered the chemical structure of the drug [45,61].

The average particle size and PDI of the gold nanoparticles and CPG-NPs are given in Table 1. The particle size increased from 50.39 ± 2.36 nm to 113 ± 4.20 nm due to the PVP coating. In addition to the drug coating, the particle size was increased to 126 ± 2.35 nm (Supplementary Materials Figures S3 and S4). This indicated that the drug was coated on the gold nanoparticles, leading to increased particle sizes. The ZP was found to decrease from −33.45 ± 3.51 mV to −21.5 ± 2.14 mV (Supplementary Materials Figure S4) with conjugation, which could be attributed to hydrogen bonding and a reduction in the charged groups, as shown in the IR spectroscopy results. The ZP indicated the electrostatic particulate stability [62,63].

### 4.2. Characterization of CPG-NP-Coated Suture

Alginate is a polysaccharide that comprises homopolymer -D-mannuronic acid and -L-guluronic acid regions. It is commonly used as an immune isolation membrane for transplantation and as an enzyme immobilizer [64]. As a result, the CPG-NPs were immobilized before being coated onto the sutures. Immobilization creates a chemically inert and biocompatible system with great temperature tolerance while maintaining pharmacological activity. A slurry dipping approach in aseptic conditions under laminar flow was used to coat the sutures with alginate CPG-NPs, and a coating time of 90 min was found to be optimal for the drug content (Table 2).

#### 4.2.1. Surface Morphology

**Figure 2:**
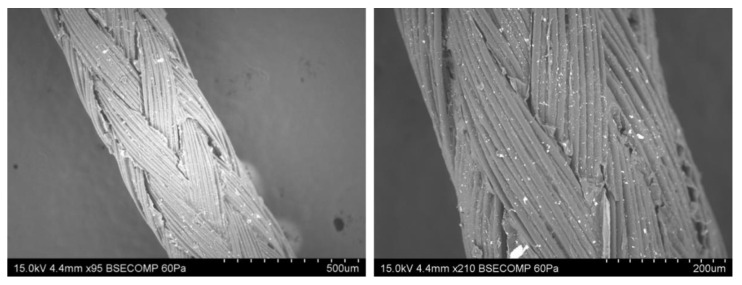
SEM images of CPG-NP-coated sutures at 95× and 210×.

SEM was used to examine the topological characteristics of the synthesized blank G-NPs, CPG-NPs, and coated sutures (Figure 2). The G-NPs and CPG-NPs showed smooth surfaces with spherical shapes (Supplementary Materials Figure S4) [51]. In the case of the CPG-NP-coated sutures, nanoparticles were coated uniformly on the surfaces of the sutures, as shown in Figure 2.

#### 4.2.2. In Vitro Release Study

**Figure 3:**
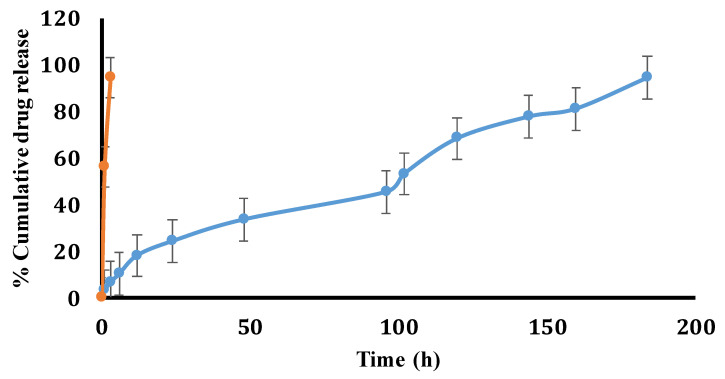
In vitro release profile of CPG-NP-coated sutures (blue lines) and CPH-coated sutures (orange) at different time intervals.

The in vitro release of drugs from CPH-coated and CPG-NP-coated sutures was conducted (Figure 3). Ciprofloxacin-coated sutures released the maximum amount of the drug within 3 h, whereas a CPG-NP-coated suture released the drug for up to 180 h. The controlled release of the drug from the CPG-NPs may have been due to hydrogen bonding between the drug and gold nanoparticles via PVP. Furthermore, the ionotropic rigidization using alginate and calcium chloride to form a hardened coat onto the suture may have further prolonged the drug release. When localized surgical site protection is necessary for an adequate period of wound healing, a longer duration of medication release is always advantageous.

#### 4.2.3. Measurement of Mechanical Properties (TS, E/B)

The mechanical qualities of the suture are crucial because they determine how easily the physician can tie a knot in surgery. The diameter of the marketed suture was 0.371 ± 0.18 mm with a TS of 3.92 ± 0.154 kg/m^2^, while the coated suture diameter was 0.399 ± 0.33 mm with a TS of 3.90 ± 0.150 kg/m^2^. The TS of the coated suture was not affected, and at the same time, the elongation at break was 32.34 ± 2.39% for uncoated sutures and approximately 30.78 ± 3.16% for drug-coated sutures. Hence, it was established that the mechanical properties of the sutures were unaffected by the coating.

#### 4.2.4. Stability Studies

Stability studies were performed under different temperature and humidity conditions, as shown in Table 3. The sutures were evaluated for their drug content and cumulative drug release (%). A similarity factor (f2) was calculated to determine the difference in the drug release profiles [65]. It was found that no significant difference was present in terms of its drug content and cumulative drug release when the suture was stored at 2–8 °C, 25 ± 2 °C/60 ± 5% RH, and 40 ± 2 °C/75 ± 5% RH. A similarity factor greater than 90% indicates high similarity between the release profiles on the 30th day compared with drug release on the 0th day, which indicated that the CPG-NP-loaded sutures were highly stable during their storage.

#### 4.2.5. Antibacterial Activity

**Figure 4:**
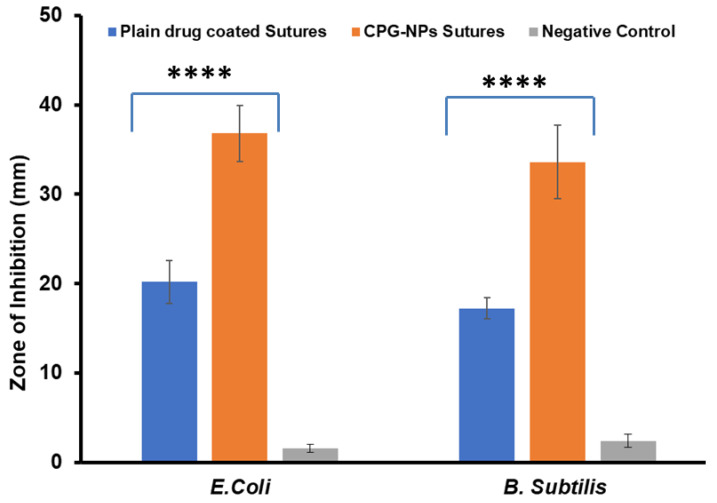
Comparison of antibacterial properties. Zone of inhibition (mm) of plain drug-coated (CPH) sutures and CPG-NP sutures on *B. subtilis and E. coli* (*n* = 3). **** *p* < 0.0001 plain drug-coated sutures (CPH) vs. CPG-NP-coated sutures.

The primary goal of coating sutures with drug-loaded gold nanoparticles was to limit bacterial development, allowing for healing and SSI prevention. As shown by the results in terms of zone of inhibition, the antibiotic released from the coated sutures hindered the development of bacteria. Antibacterial assays were performed on Gram-negative *(Escherichia coli*) and Gram-positive *(Bacillus subtilis*) microorganisms, and the zone of inhibition is shown in Figure 4. An approximately two-fold increase in the zone of inhibition was observed with CPG-NP-loaded sutures compared with plain drug sutures in both organisms. The results obtained from the study displayed the greater efficacy of the CPG-NP-coated sutures than plain drug-coated sutures, and the difference was statistically significant. This may have been due to the increased penetration of the CPG-NPs into the bacterial cell membrane and its superior ability to accumulate CPH intracellularly and cause bacterial cell death. In addition, because of the presence of the NPs, the increased surface area of NPs carried a high amount of drug on its surface, which led to an increased local CPH concentration at the site of the bacterium–particle contact. This result showed the enhanced antibacterial activity of CPG-NPs compared with free CPH. Increased potency for 72 h may also be due to the controlled drug release from the conjugated carriers enhancing the duration of inhibition of bacterial colonization compared with plain drug-coated sutures [40,43]. However, while G-NPs themselves do not have any antimicrobial activity, they may act as drug carriers [66].

#### 4.2.6. Hemolysis

According to ASTM recommendations (ASTM; F 756–08), hemolysis testing is a convenient, uniform, and repeatable method for determining biomaterial compatibility. According to the guidelines, materials were divided into three categories based on their hemolytic index (hemolysis percent). A material is described as very hemolytic if the proportion of hemolysis is greater than 5%. If the rate of hemolysis is between 5% and 2%, it is considered slightly hemolytic, and if it is less than 2%, it is called nonhemolytic. Hemolysis studies help to verify the suitability of biomaterials that have been coated with blood cells, as their toxicity must be determined to demonstrate their safety profile. CPG-NP-coated sutures, like uncoated sutures, fell into the nonhemolytic category, explaining their biocompatibility (Table 4).

#### 4.2.7. Histopathology

**Figure 5:**
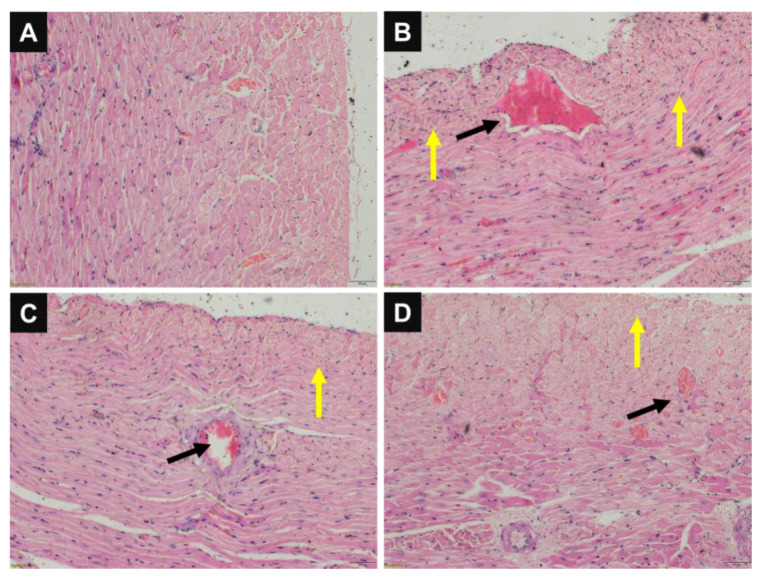
Histopathology studies depicting a (**A**) negative control, (**B**) plain suture, (**C**) plain drug (CPH)-coated suture, and (**D**) CPG-NP-coated suture. The black arrows indicate erythema and the yellow arrows specify the granulation tissue. Note: group I (negative control), group II (plain suture), group III (CPH-coated suture), and group IV (CPG-NP-coated suture); 10× magnification and the scale bar is 50 µm.

Wound healing is determined by the infiltration of inflammatory cells, collagen, fibroblast levels, pattern of granulation, etc. In Figure 5, the histopathology of the intestine at the site of anastomosis is presented. The study revealed that uncoated or plain sutures (group II) showed prominent erythema, adhesions, hemorrhage, and granulation, which indicated improper healing at the site of the surgical anastomosis. However, in the groups where CPH-coated and CPG-NP-coated sutures were used, proper healing with fewer signs of adhesions, infiltration of inflammatory cells, and granulation was observed when compared with the plain sutures. The histological scores are given in Table 5. This can be due to the well-known anti-inflammatory property of gold and the antibacterial property of ciprofloxacin.

The histological scores (Table 5) show the average inflammatory cell infiltration, as well as the fibroblast deposition. When compared with the negative control, the ratings for inflammation were significantly lower with coated sutures, which could be attributed to the sustained release from gold nanoparticles and the antibacterial property of ciprofloxacin. From the above findings, we concluded that the CPG-NP-coated sutures did not produce any sensitization reactions and can be a viable option for use. From the histopathology images (Figure 5) of various tissues, it was found that hemorrhage occurred only with plain drug-coated sutures but not with CPG-NP-coated sutures, which correlated with the hemolysis study.

## 5. Conclusions

In conclusion, wet-solvent precipitation was used to make ciprofloxacin colloidal gold NPs using PVP, along with a cross-linked alginate coating to rigidize the coat. To improve the antibacterial property of the sutures, the produced ciprofloxacin gold nanoparticles were layered on the surface of the sutures using a dipping approach. The coated sutures were found to have antibacterial action against both Gram-negative and Gram-positive bacteria with medication release. Coated sutures repaired tissue faster in vivo than uncoated sutures. Finally, it was ascertained that the drug-coated sutures were more effective at preventing SSIs. However, this was a proof-of-concept study designed for the treatment of surgical site infections (SSIs) due to Escherichia coli (a Gram-negative bacteria) and Bacillus subtilis (a Gram-positive bacteria). Here, we investigated antibacterial activity with a limited number of microorganisms. The most isolated organisms in surgical site infections are S. aureus, coagulase-negative staphylococci, Enterococcus spp., and Escherichia coli. An increasing number of SSIs are attributable to antibiotic-resistant pathogens, such as methicillin-resistant S. aureus (MRSA) or Candida albicans [67,68,69]. To address these challenges, extensive antibacterial and in vivo studies are required to understand the impact of antibiotic-drug–NP-coated sutures during the treatment of SSIs, which are currently ongoing in our laboratories.

## Figures and Tables

**Table 1:** Particle size, PDI, and ZP of the given formulations (*n* = 3).

S. No.	Formulation	Mean Particle Size (nm)	PDI	ZP (mV)
1	G-NPs	50.39 ± 5.36	0.143 ± 0.04	−33.45 ± 3.51
2	PG-NPs	113.6 ± 8.20	0.168 ± 0.05	−29.21 ± 1.84
3	CPG-NPs	126.2 ± 13.35	0.134 ± 0.03	−21.50 ± 2.14

**Table 2:** Optimization of the contact time for drug loading on sutures (*n* = 3).

S. No.	Formulation	Time (min)	Weight of Uncoated Suture (mg)	Weight of Coated Suture after Coating (mg)	Assay of Drug Loading on Suture (µg)
				CPG-NPs	CPG-NPs
1	F1	30	30.00 ± 0.25	37.23 ± 1.52	467.70 ± 25.48
2	F2	60	30.00 ± 0.36	43.32 ± 2.33	899.61 ± 8.23
3	F3	90	30.00 ± 0.16	47.45 ± 1.56	1171.35 ± 10.69
4	F4	120	30.00 ± 0.42	46.92 ± 2.31	987.23 ± 18.12

**Table 3:** Stability study of ciprofloxacin-conjugated gold-nanoparticle-loaded sutures (*n* = 3). Data are represented as the mean ± SD.

Formulation	Storage Conditions	Assay (%)		Cumulative Drug Release (%)		Similarity Factor (f2) b/w 0th and 30th Day
		0 days	30 days	0 days	30 days	
CPG-NP-conjugated sutures	25 ± 2 °C/60 ± 5% RH	88.53 ± 3.35	83.47 ± 5.63	94.57 ± 2.23	95.19 ± 4.57	93.63 ± 2.25
	40 ± 2 °C/75 ± 5% RH	88.53 ± 3.35	80.26 ± 2.79	94.57 ± 2.23	98.94 ± 3.27	92.21 ± 3.45
	2–8 °C	88.53 ± 3.35	87.69 ± 4.23	94.57 ± 2.23	94.19 ± 5.25	95.53 ± 4.13

**Table 4:** Hemolysis of plain marketed suture, plain drug-loaded suture, and CPG-NP loaded suture (*n* = 3).

S. No.	Suture	Hemolysis (%) Mean ± SD
1	Plain marketed suture	1.2 ± 0.19
2	CPH-coated suture	8.45 ± 0.15
3	CPG-NP-loaded suture	1.56 ± 0.24

**Table 5:** Histopathology scoring studies for all treatment groups (*n* = 8).

Parameter	Group I (Negative Control)	Group II (Plain Suture)	Group III (CPH-Coated Suture)	Group IV (CPG-NP-Coated Suture)
Hemorrhage	0	4	1	0
Maceration	0	0	0	0
Erythema	0	3	0	0
Necrosis	0	2	2	1
Adherence	0	1	1	1
Granulation tissue	0	3	1	1
Epithelialization	0	0	0	0
Total score	0	13	5	3

## Data Availability

Data available within the article, additional data available from S.S. upon reasonable request.

## Supplementary Materials

The following supporting information can be downloaded at: https://www.mdpi.com/article/10.3390/pharmaceutics14102130/s1, Figure S1: UV–Vis spectra; Figure S2: IR overlay; Figure S3: Particle size and zeta potential; Figure S4: SEM image; Method S1: Preparation of standard curve of ciprofloxacin hydrochloride; Figure S5. Standard curve.

## References

[1] Devassy G., Ramachandran R., Jeena K., Junnuthula V.R., Gopinatha V.K., Manju C., Manohar M., Nair S.V., Raghavan S.C., Koyakutty M. (2019). Simultaneous release of two drugs from polymer nano-implant inhibits recurrence in glioblastoma spheroids. Precis. Nanomed..

[2] Ramachandran R., Junnuthula V.R., Gowd G.S., Ashokan A., Thomas J., Peethambaran R., Thomas A., Unni A.K.K., Panikar D., Nair S.V. (2017). Theranostic 3-Dimensional nano brain-implant for prolonged and localized treatment of recurrent glioma. Sci. Rep..

[3] Schmitt E., Epstein M. (1973). Method of Attaching Surgical Needles to Multifilament Polyglycolic Acid Absorbable Sutures. US Patent.

[4] Martin-Bates A. (2008). Tying all together. Trauma.

[5] Chen C.K., Lee M.C., Lin Z.I., Lee C.A., Tung Y.C., Lou C.W., Law W.C., Chen N.T., Lin K.Y.A., Lin J.H. (2019). Intensifying the Antimicrobial Activity of Poly[2-(tert-butylamino)ethyl Methacrylate]/Polylactide Composites by Tailoring Their Chemical and Physical Structures. Mol. Pharm..

[6] Hoque J., Prakash R.G., Paramanandham K., Shome B.R., Haldar J. (2017). Biocompatible injectable hydrogel with potent wound healing and antibacterial properties. Mol. Pharm..

[7] Lin Z.I., Tsai H.L., Liu G.L., Lu X.H., Cheng P.W., Chi P.L., Wang C.K., Tsai T.H., Wang C.C., Yang J.H.C. (2022). Preparation of CO_2_-Based Cationic Polycarbonate/Polyacrylonitrile Nanofibers with an Optimal Fibrous Microstructure for Antibacterial Applications. Macromol. Biosci..

[8] Inglis B. (1965). a History of Medicine. Lancet.

[9] Mellinghoff S.C., Vehreschild J.J., Liss B.J., Cornely O.A. (2018). Epidemiology of surgical site infections with Staphylococcus aureus in Europe: Protocol for a retrospective, multicenter study. JMIR Res. Protoc..

[10] Aliyu S., Furuya Y., Larson E. (2019). Risk of subsequent health care–associated infection among patients with a bloodstream infection present on hospital admission. Am. J. Infect. Control.

[11] Elmously A., Gray K.D., Michelassi F., Afaneh C., Kluger M.D., Salemi A., Watkins A.C., Pomp A. (2019). Operating Room Attire Policy and Healthcare Cost: Favoring Evidence over Action for Prevention of Surgical Site Infections. J. Am. Coll. Surg..

[12] Hsu H.E., Kawai A., Wang R., Jentzsch M.S., Rhee C., Horan K., Jin R., Goldmann N., Lee G.M. (2018). The Impact of the Medicaid Healthcare-Associated Condition Program on Mediastinitis Following Coronary Artery Bypass Graft. Infect. Control Hosp. Epidemiol..

[13] Hranjec T., Swenson B.R., Sawyer R.G. (2010). Surgical site infection prevention: How we do it. Surg. Infect..

[14] Mingmalairak C. (2011). Antimicrobial sutures: New strategy in surgical site infections. Sci. Against Microb. Pathog. Commun. Curr. Res. Technol. Adv..

[15] Okoro H.K., Ige J.O., Iyiola O.A., Pandey S., Lawal I.A., Zvinowanda C., Ngila C.J. (2017). Comprehensive reviews on adverse health effects of human exposure to endocrine-disrupting chemicals. Fresenius Environ. Bull..

[16] Pycke B.F.G., Geer L.A., Dalloul M., Abulafia O., Jenck A.M., Halden R.U. (2014). Human fetal exposure to triclosan and triclocarban in an urban population from Brooklyn, New York. Environ. Sci. Technol..

[17] Dennis C., Sethu S., Nayak S., Loganathan M., Morsi Y., Manivasagam G. (2016). Suture materials—Current and emerging trends. J. Biomed. Mater. Res.—Part A.

[18] Brooks B.D., Brooks A.E., Grainger D.W. (2013). Antimicrobial medical devices in preclinical development and clinical use. Biomaterials Associated Infection: Immunological Aspects and Antimicrobial Strategies.

[19] Bhusal P., Harrison J., Sharma M., Jones D.S., Hill A.G., Svirskis D. (2016). Controlled release drug delivery systems to improve post-operative pharmacotherapy. Drug Deliv. Transl. Res..

[20] Bernhardt M. (2016). Suture materials. Skinmed.

[21] Chu C.C. (1997). Classification and General Characteristics of Suture Materials. Wound Closure Biomaterials and Devices.

[22] Bawa R., Audette G.F., Rubinstein I. (2016). Handbook of Clinical Nanomedicine: Nanoparticles, Imaging, Therapy and Clinical Applications.

[23] Marcato P.D., Durán N. (2008). New aspects of nanopharmaceutical delivery systems. J. Nanosci. Nanotechnol..

[24] Su H., Wang Y., Gu Y., Bowman L., Zhao J., Ding M. (2018). Potential applications and human biosafety of nanomaterials used in nanomedicine. J. Appl. Toxicol..

[25] Sarkar A., Junnuthula V., Dyawanapelly S. (2021). Ocular therapeutics and molecular delivery strategies for neovascular age-related macular degeneration (Namd). Int. J. Mol. Sci..

[26] Sarkar A., Sodha S.J., Junnuthula V., Kolimi P., Dyawanapelly S. (2022). Novel and investigational therapies for wet and dry age-related macular degeneration. Drug Discov. Today.

[27] Pailla S.R., Talluri S., Rangaraj N., Ramavath R., Challa V.S., Doijad N., Sampathi S. (2019). Intranasal Zotepine Nanosuspension: Intended for improved brain distribution in rats. DARU J. Pharm. Sci..

[28] Jain A.S., Pawar P.S., Sarkar A., Junnuthula V., Dyawanapelly S. (2021). Bionanofactories for green synthesis of silver nanoparticles: Toward antimicrobial applications. Int. J. Mol. Sci..

[29] Gold K., Slay B., Knackstedt M., Gaharwar A.K. (2018). Antimicrobial Activity of Metal and Metal-Oxide Based Nanoparticles. Adv. Ther..

[30] Dykman L., Khlebtsov N. (2012). Gold nanoparticles in biomedical applications: Recent advances and perspectives. Chem. Soc. Rev..

[31] Zhang X. (2015). Gold Nanoparticles: Recent Advances in the Biomedical Applications. Cell Biochem. Biophys..

[32] Lohse S.E., Eller J.R., Sivapalan S.T., Plews M.R., Murphy C.J. (2013). A simple millifluidic benchtop reactor system for the high-throughput synthesis and functionalization of gold nanoparticles with different sizes and shapes. ACS Nano.

[33] Ridolfo R., Tavakoli S., Junnuthula V., Williams D.S., Urtti A., Van Hest J.C.M. (2021). Exploring the Impact of Morphology on the Properties of Biodegradable Nanoparticles and Their Diffusion in Complex Biological Medium. Biomacromolecules.

[34] Rastogi L., Kora A.J., Arunachalam J. (2012). Highly stable, protein capped gold nanoparticles as effective drug delivery vehicles for amino-glycosidic antibiotics. Mater. Sci. Eng. C.

[35] Liong M., Lu J., Kovochich M., Xia T., Ruehm S.G., Nel A.E., Tamanoi F., Zink J.I. (2008). Multifunctional inorganic nanoparticles for imaging, targeting, and drug delivery. ACS Nano.

[36] Pailla S., Sampathi S., Junnuthula V., Maddukuri S., Dodoala S., Dyawanapelly S. (2022). Brain-Targeted Intranasal Delivery of Zotepine Microemulsion: Pharmacokinetics and Pharmacodynamics. Pharmaceutics.

[37] Zhu G., Lin Z.H., Jing Q., Bai P., Pan C., Yang Y., Zhou Y., Wang Z.L. (2013). Toward large-scale energy harvesting by a nanoparticle-enhanced triboelectric nanogenerator. Nano Lett..

[38] Khairnar S.V., Pagare P., Thakre A., Nambiar A.R., Junnuthula V., Abraham M.C., Kolimi P., Nyavanandi D., Dyawanapelly S. (2022). Review on the Scale-Up Methods for the Preparation of Solid Lipid Nanoparticles. Pharmaceutics.

[39] Xia Q., Li H., Xiao K. (2016). Factors Affecting the Pharmacokinetics, Biodistribution and Toxicity of Gold Nanoparticles in Drug Delivery. Curr. Drug Metab..

[40] Li X., Robinson S.M., Gupta A., Saha K., Jiang Z., Moyano D.F., Sahar A., Riley M.A., Rotello V.M. (2014). Functional gold nanoparticles as potent antimicrobial agents against multi-drug-resistant bacteria. ACS Nano.

[41] Albanese A., Tang P.S., Chan W.C.W. (2012). The effect of nanoparticle size, shape, and surface chemistry on biological systems. Annu. Rev. Biomed. Eng..

[42] Hajipour M.J., Fromm K.M., Ashkarran A.A., de Aberasturi D.J., de Larramendi I.R., Rojo T., Serpooshan V., Parak W.J., Mahmoudi M. (2012). Antibacterial properties of nanoparticles. Trends Biotechnol..

[43] Allahverdiyev A.M., Kon K.V., Abamor E.S., Bagirova M., Rafailovich M. (2011). Coping with antibiotic resistance: Combining nanoparticles with antibiotics and other antimicrobial agents. Expert Rev. Anti. Infect. Ther..

[44] Ramalingam V., Varunkumar K., Ravikumar V., Rajaram R. (2018). Target delivery of doxorubicin tethered with PVP stabilized gold nanoparticles for effective treatment of lung cancer. Sci. Rep..

[45] Gangwar R.K., Dhumale V.A., Kumari D., Nakate U.T., Gosavi S.W., Sharma R.B., Kale S.N., Datar S. (2012). Conjugation of curcumin with PVP capped gold nanoparticles for improving bioavailability. Mater. Sci. Eng. C.

[46] Elveren B., Yildiz Ü.H., Yildiz A.A. (2018). Utilization of near ir absorbing gold nanocolloids by green synthesis. the Mater. Sci. Forum.

[47] Navarrete J., Siefe C., Alcantar S., Belt M., Stucky G.D., Moskovits M. (2018). Merely Measuring the UV-Visible Spectrum of Gold Nanoparticles Can Change Their Charge State. Nano Lett..

[48] Espinosa C.E., Guo Q., Singh V., Behrens S.H. (2010). Particle charging and charge screening in nonpolar dispersions with nonionic surfactants. Langmuir.

[49] Junnuthula V., Kolimi P., Nyavanandi D., Sampathi S., Vora L.K., Dyawanapelly S. (2022). Polymeric Micelles for Breast Cancer Therapy: Recent Updates, Clinical Translation and Regulatory Considerations. Pharmaceutics.

[50] Junnuthula V., Sadeghi Boroujeni A., Cao S., Tavakoli S., Ridolfo R., Toropainen E., Ruponen M., van Hest J.C.M., Urtti A. (2021). Intravitreal polymeric nanocarriers with long ocular retention and targeted delivery to the retina and optic nerve head region. Pharmaceutics.

[51] Sunitha S., Adinarayana K., Sravanthi R.P., Sonia G., Nagarjun R., Pankaj T., Veerabhadra S.C., Sujatha D. (2017). Fabrication of Surgical Sutures Coated with Curcumin Loaded Gold Nanoparticles. Pharm. Anal. Acta.

[52] Nazem-Bokaee H., Fallahianbijan F., Chen D., O’Donnell S.M., Carbrello C., Giglia S., Bell D., Zydney A.L. (2018). Probing pore structure of virus filters using scanning electron microscopy with gold nanoparticles. J. Membr. Sci..

[53] Sampathi S., Prajapati S., Junnuthula V., Dyawanapelly S. (2022). Pharmacokinetics and Anti-Diabetic Studies of Gliclazide Nanosuspension. Pharmaceutics.

[54] Marques M.R.C., Loebenberg R., Almukainzi M. (2011). Simulated biological fluids with possible application in dissolution testing. Dissolution Technol..

[55] Abullais S.S., Alqahtani N.A., Alkhulban R.M., Alamer S.H., Khan A.A., Pimple S. (2020). In-vitro evaluation of commonly used beverages on tensile strength of different suture materials used in dental surgeries. Medicine.

[56] Thakkar S., Sharma D., Misra M. (2018). Comparative evaluation of electrospraying and lyophilization techniques on solid state properties of Erlotinib nanocrystals: Assessment of In-vitro cytotoxicity. Eur. J. Pharm. Sci..

[57] Man R.W.Y., Li C.H., MacLean M.W.A., Zenkina O.V., Zamora M.T., Saunders L.N., Rousina-Webb A., Nambo M., Crudden C.M. (2018). Ultrastable gold nanoparticles modified by bidentate N-Heterocyclic Carbene Ligands. J. Am. Chem. Soc..

[58] Klančnik A., Piskernik S., Jeršek B., Možina S.S. (2010). Evaluation of diffusion and dilution methods to determine the antibacterial activity of plant extracts. J. Microbiol. Methods.

[59] Ehrlich H.P., Tarver H., Hunt T.K. (1973). Effects of vitamin A and glucocorticoids upon inflammation and collagen synthesis. Ann. Surg..

[60] Ehrlich H.P., Hunt T.K. (1968). Effects of cortisone and vitamin A on wound healing. Ann. Surg..

[61] Dubas S.T., Wacharanad S., Potiyaraj P. (2011). Tunning of the antimicrobial activity of surgical sutures coated with silver nanoparticles. Colloids Surfaces A Physicochem. Eng. Asp..

[62] Heurtault B., Saulnier P., Pech B., Proust J.E., Benoit J.P. (2003). Physico-chemical stability of colloidal lipid particles. Biomaterials.

[63] El Badawy A.M., Luxton T.P., Silva R.G., Scheckel K.G., Suidan M.T., Tolaymat T.M. (2010). Impact of environmental conditions (pH, ionic strength, and electrolyte type) on the surface charge and aggregation of silver nanoparticles suspensions. Environ. Sci. Technol..

[64] Hertzberg S., Kvittingen L., Anthonsen T., Skjåk-Bræk G. (1992). Alginate as immobilization matrix and stabilizing agent in a two-phase liquid system: Application in lipase-catalysed reactions. Enzym. Microb. Technol..

[65] Gao Y., Zu H., Zhang J. (2011). Enhanced dissolution and stability of adefovir dipivoxil by cocrystal formation. J. Pharm. Pharmacol..

[66] Burygin G.L., Khlebtsov B.N., Shantrokha A.N., Dykman L.A., Bogatyrev V.A., Khlebtsov N.G. (2009). On the enhanced antibacterial activity of antibiotics mixed with gold nanoparticles. Nanoscale Res. Lett..

[67] Owens C.D., Stoessel K. (2018). Surgical site infections: Epidemiology, microbiology and prevention. J. Hosp. Infect..

[68] Mingoia M., Conte C., Di Rienzo A., Dimmito M.P., Marinucci L., Magi G., Turkez H., Cufaro M.C., Del Boccio P., Di Stefano A. (2022). Synthesis and Biological Evaluation of Novel Cinnamic Acid-Based Antimicrobials. Pharmaceuticals.

[69] Vieira D., Angel S.N., Honjol Y., Masse M., Gruenheid S., Harvey E.J., Merle G. (2022). Engineering surgical stitches to prevent bacterial infection. Sci. Rep..

