# Supplementary material for: Development of Biocompatible Ciprofloxacin–Gold Nanoparticle Coated Sutures for Surgical Site Infections
Source: Pharmaceutics. 2022 Oct 7;14(10):2130. doi: 10.3390/pharmaceutics14102130 (PMC9608442; doi:10.3390/pharmaceutics14102130)
Supplement: Supplementary file 1 [file pharmaceutics-14-02130-s001.zip › pharmaceutics-1897389-supplementary.pdf]

Supplementary Materials: Development of Biocompatible Ciprofloxacin–Gold Nanoparticle Coated Sutures for Surgical Site Infections

Sunitha Sampathi, Pankaj Kumar Tiriya, Sujatha Dodoala, Vijayabhaskarreddy Junnuthula and Sathish Dyawanapelly

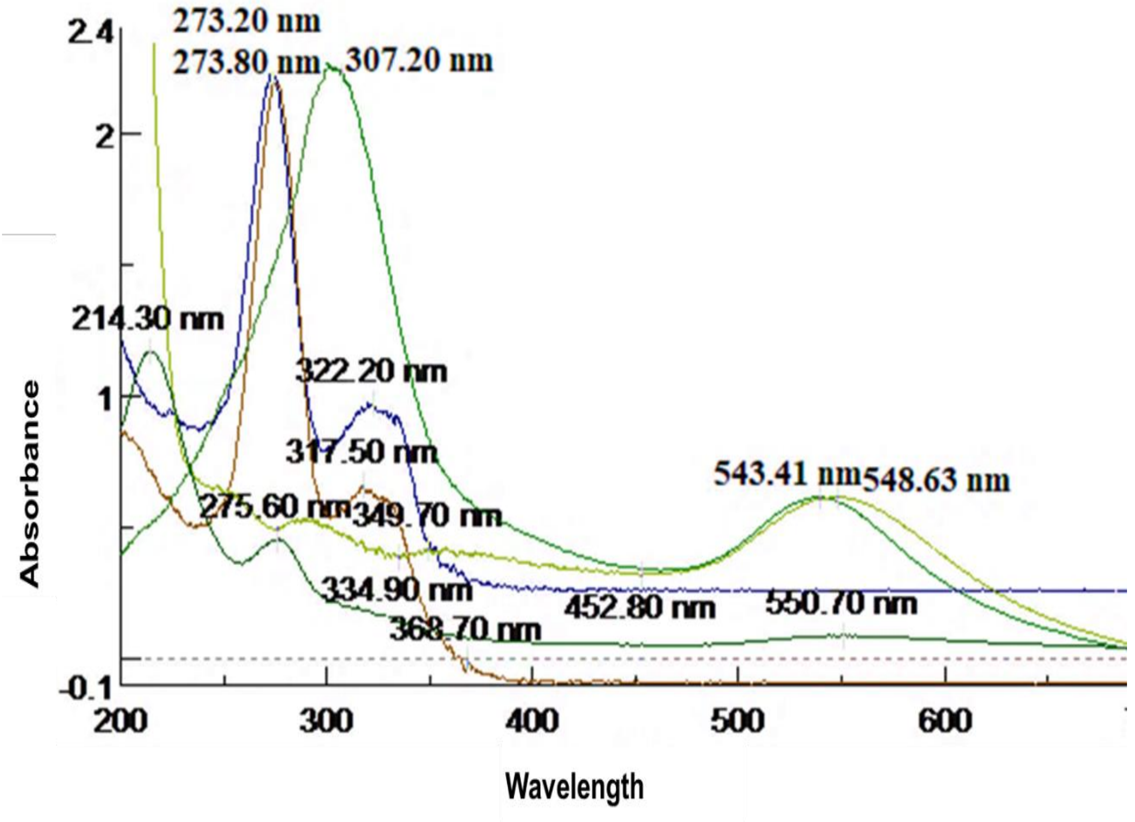

Figure S1. UV–Vis spectra of 1) G-NP (Black band) 2) PG-NP (yellow band) 3) CPG-NP (Dark green band) 4) CPH (dark blue band) 5) PVP capped CPH (Red band).

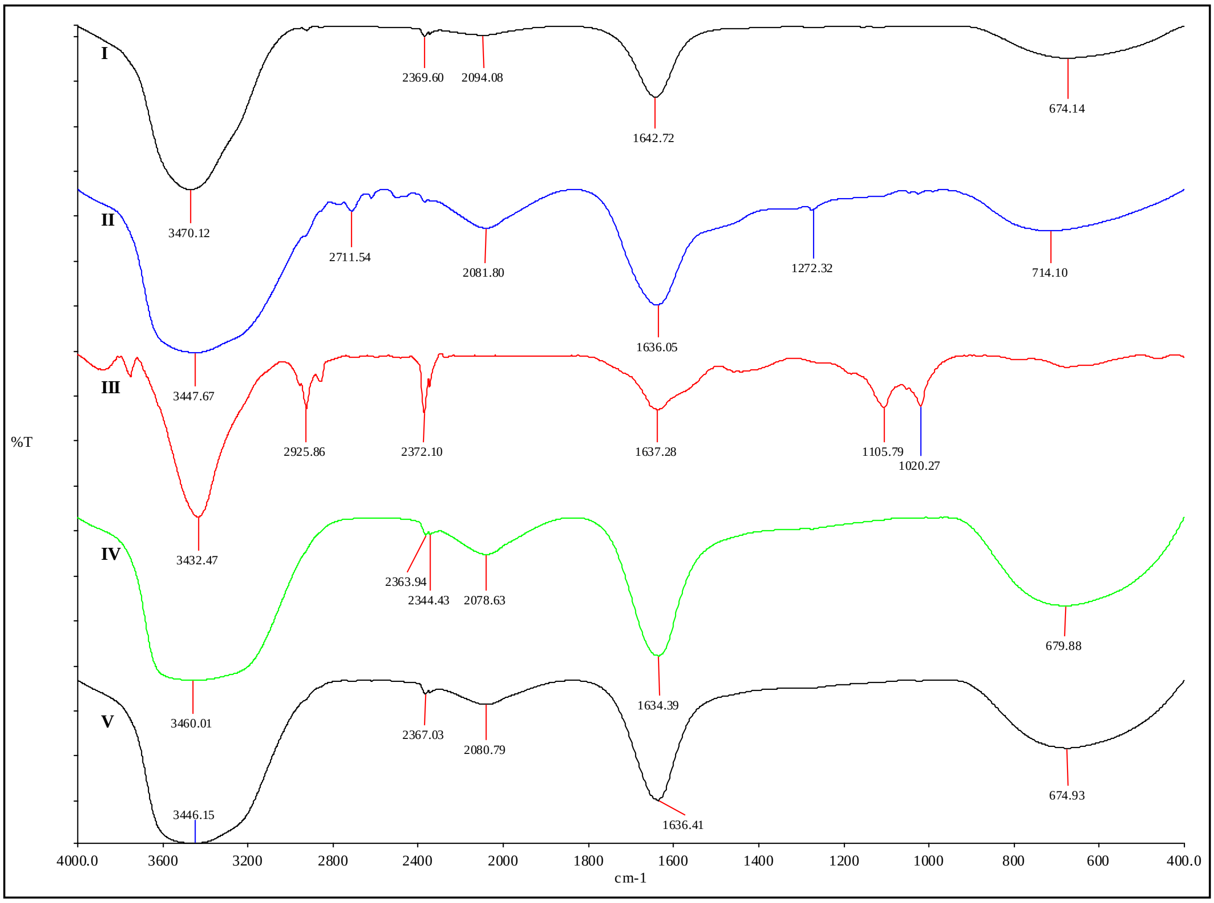

Figure S2. IR overlay of G-NP, CPH, PG-NP, physical mixture and CPG-NP (I-V order).

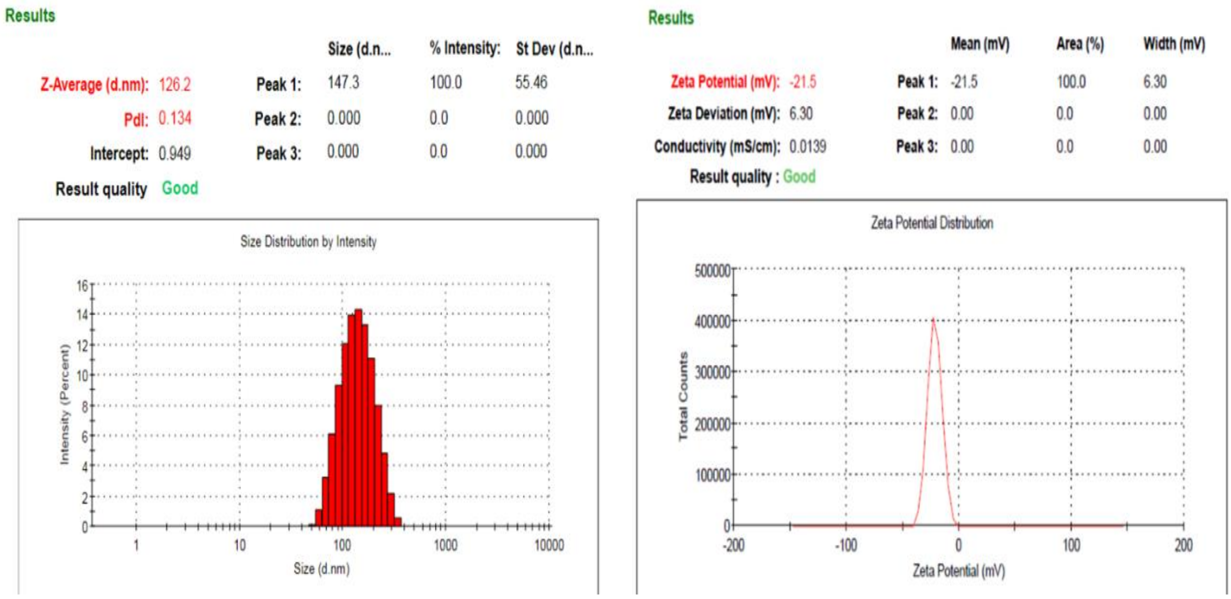

Figure S3. Particle size and zeta potential of optimized CPG-NP.

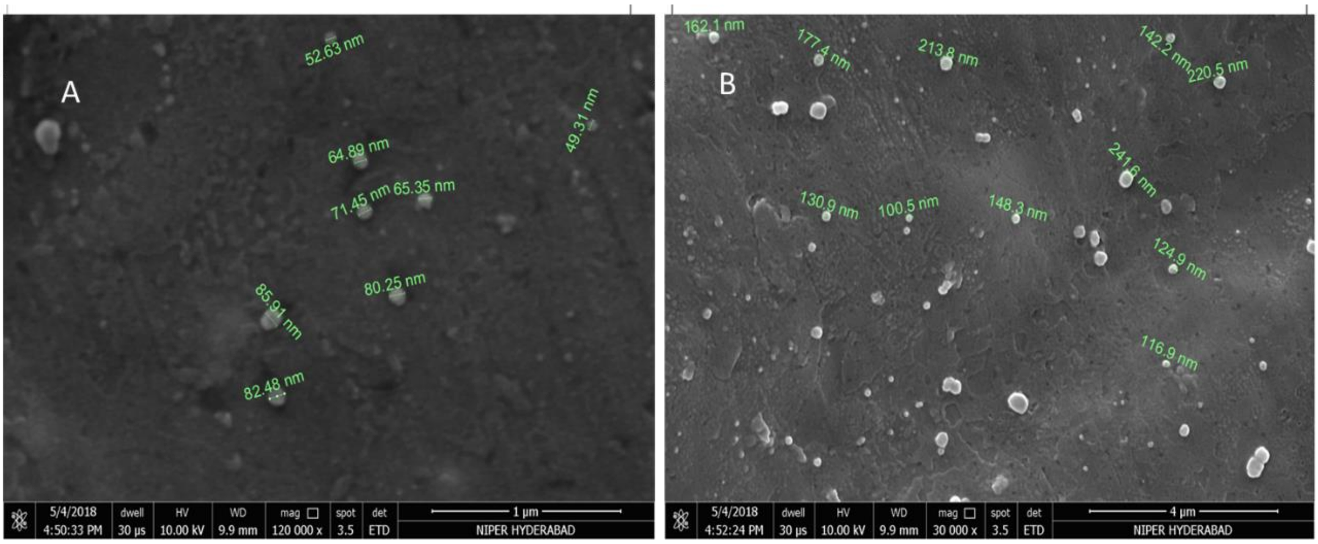

Figure S4. SEM image of plain G-NP (A) and CPG-NP (B) respectively.

Method S1: Preparation of standard curve of ciprofloxacin hydrochloride in Deionized water by UV-visible Spectrophotometer.

Ciprofloxacin hydrochloride primary stock solution of 1000 µg/mL was prepared in deionized water by dissolving 10 mg of drug in 10 mL of deionized water. From the primary stock, a secondary stock solution of 100 µg/mL was prepared. Aliquot of 1 mL was pipette out into a 10 mL volumetric flask and volume was made up to 10 mL with water to get concentration of 10 µg/mL. Sample was scanned using water as a blank within the wavelength range of 200 nm to 800 nm. The wavelength at which maximum absorption obtained was noted as λmax**.**

From secondary stock solution (100 µg/mL) aliquots of 2 mL, 3 mL, 4 mL, 5 mL, 6 mL, 7 mL and 8 mL, 9 mL were pipette out into a series of 10 mL volumetric flasks and volume was made up to 10 mL with water to get concentrations in the range of 2 to 14 µg/mL. The absorbance of the resulting solution was then measured at 273 nm using UV-Visible spectrophotometer against respective solvent as blank. The standard curve was obtained by plotting absorbance versus concentration in µg/mL.

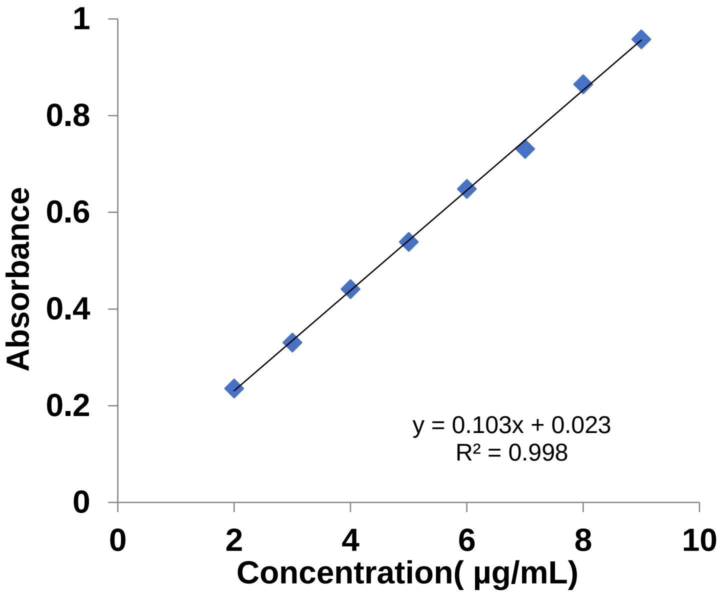

Figure S5. Standard curve of ciprofloxacin in Deionized Water.
